# External Negative Pressure Drainage of the Pancreatic Duct in Pancreatogastrostomy Following Pylorus-Preserving Pancreaticoduodenectomy—Feasibility and Technique

**DOI:** 10.3389/fsurg.2021.754288

**Published:** 2021-11-17

**Authors:** H. C. Albrecht, C. Amling, C. Menenakos, S. Gretschel

**Affiliations:** ^1^Department of General, Visceral, Thoracic and Vascular Surgery, Faculty of Health Brandenburg, Brandenburg Medical School, University Hospital Neuruppin, Neuruppin, Germany; ^2^Department of General, Visceral, Thoracic and Vascular Surgery, Academic Teaching Hospital of Charité Medical School, Werner Forßmann Hospital Eberswalde, Eberswalde, Germany

**Keywords:** pancreaticoduodenectomy, pancreatogastrostomy, postoperative pancreatic fistula, pancreas duct drainage, soft pancreas

## Abstract

**Background:** Postoperative pancreatic fistula (POPF) is a major cause of morbidity after pancreaticoduodenectomy. There is no consensus on the best technique to protect the pancreato-enteric anastomosis and reduce the rate of POPF. This study investigated the feasibility and efficiency of external suction drainage of the pancreatic duct to improve the healing of pancreaticogastrostomy.

**Methods:** Between July 2019 and June 2021, 21 consecutive patients undergoing elective pancreaticoduodenectomy were included. In all patients we performed a pancreaticogastrostomy and inserted a negative pressure drainage into the pancreatic duct. The length and diameter of the pancreatic duct were measured and the texture of the pancreas was evaluated. The daily secretion volume and the lipase value via pancreatic duct drainage were documented. The occurrence of POPF was evaluated.

**Results:** None of the patients had drainage-related complications. In 4 patients we registered a dislocation of the drainage from the pancreas duct into the stomach. 17/21 Patients showed no signs of POPF. A biochemical leak was measured in one patient. Furthermore, 2 patients had a POPF grade B. In one patient, POPF grade C required reoperation and resection of the remnant pancreas. All 4 cases of POPF met the risk criteria soft pancreas, high volume and high lipase value in the duct drainage.

**Conclusion:** The insertion of the pancreatic duct drainage was feasible and caused no drainage-related morbidity. POPF-rate was moderate in the risk population of soft pancreas and small duct.

## Introduction

Pancreaticoduodenectomy (PD) is the gold standard in the treatment of cancer of the pancreatic head. Although perioperative mortality has decreased significantly, morbidity remains a concern as it can be as high as to 50% (1). Postoperative pancreatic fistula (POPF) is the leading cause of morbidity after PD, with reported incidence of 10 to 35%. (2). According to Daskalaki et al. 19% of fistulas are clinically irrelevant, 70.7% require conservative or interventional treatment (grade B), and severe complications occur in 8.8% (grade C) (3).

Under these terms, different methods and technical versions for the creation of a pancreatic anastomosis—pancreaticojejunostomy (PJ) or pancreaticogastrostomy (PG) have been proposed to avoid anastomotic leak with POPF. Widely used methods include the application of adhesive sealants around the anastomosis, a flap of Ligament teres, the use of transanastomotic stents, drainage and the use of various systemic pharmacological agents (4).

However, no consensus has yet been reached on the best technique to protect the pancreato-enteric anastomosis and reduce the rate of POPF. The following study investigated the feasibility and efficiency of inserting an external suction drain into the pancreatic duct to improve the healing of PG following PD. All studies investigating the impact of duct drainage so far refer only to pancreato-jejunostomy, no data are available for pancreato-gastrostomy.

## Patients And Methods

Between July 2019 and June 2021, 21 consecutive patients undergoing elective pancreaticoduodenectomy (PD) for benign or malignant pathologies of the pancreas or periampullary region were enrolled in our study.

### Surgical Technique

**Figure 1:**
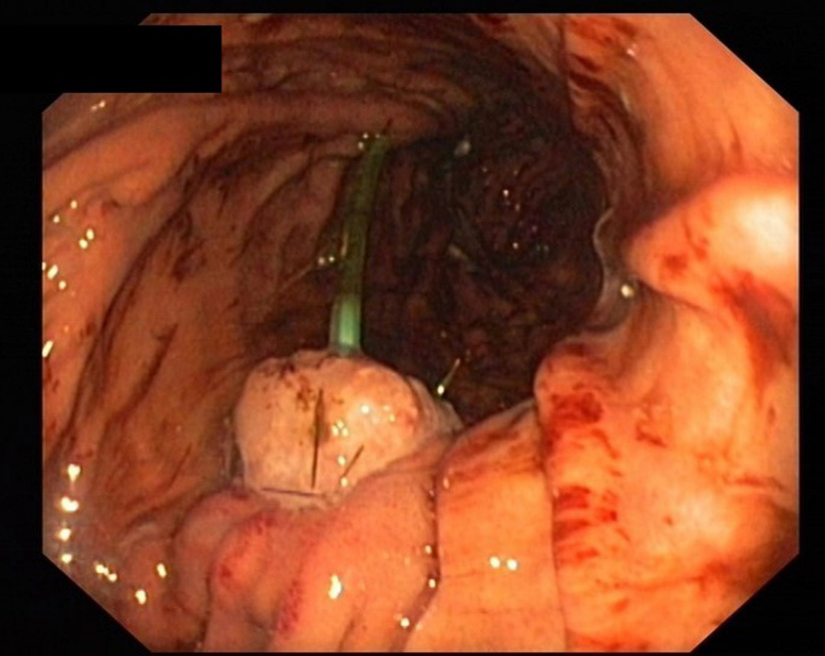
Implanted pancreatic stump with duct drainage.

**Figure 2:**
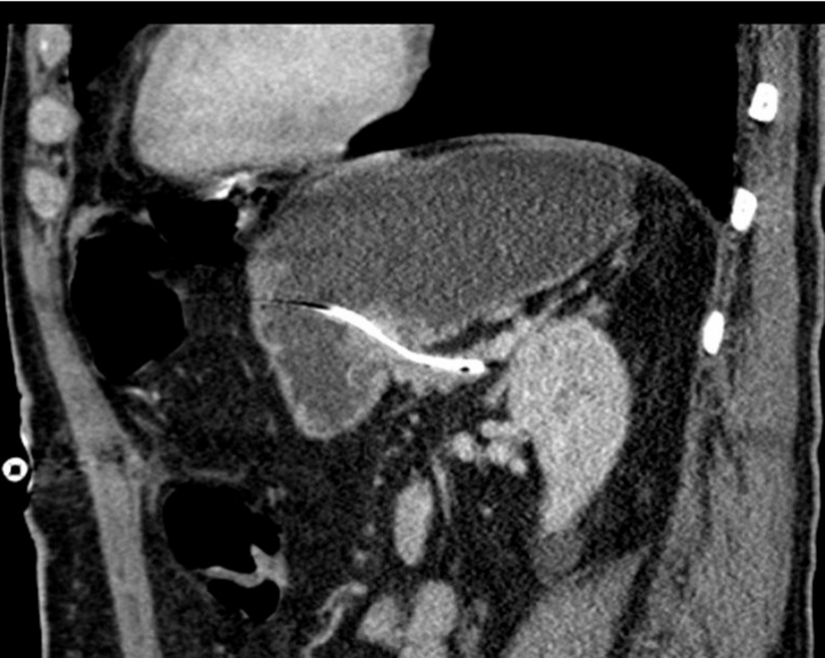
CT image of pancreatic duct drainage in the pancreatic stump and stomach.

All patients received perioperative antibiotics. After informed consent of the patient, PD was performed as a partial pancreatectomy with pylorus preservation. After resection, the length and diameter of the pancreatic duct was measured to calculate the size of the drainage. The texture of the pancreas (soft/middle/hard) was evaluated and documented by the surgeon. Pancreatic anastomosis was constructed as a pancreaticogastrostomy with the use of monofilament absorbable sutures (in two layers as purse-string seromuscular suture + single button mucosa suture). A pediatric feeding tube made of silastic polyethylene (Vygon, France) with additional lateral holes was inserted into the pancreatic duct for drainage (Figure 1). The tube was fixed to the pancreatic stump with a suture (6 ×0 Marlin® rapid, Catgut GmbH, Germany), then the tube was pulled through the ventral incision of the stomach. The incision was closed with a two-layer continuous suture, and the catheter is covered with the gastric serosomuscular layer in a length of 3 cm similar to a Witzel-fistula. The usual single loop reconstruction with bilio-jejunal and pyloro-jejunal anastomosis was then completed. Finally, the drainage of the pancreatic duct was then externalized through a stab incision in the ventral abdominal wall and fixed to the skin to prevent catheter migration. A CT scan of the pancreatic drainage is shown in Figure 2. The drainage was connected to a negative pressure suction system (pri-aktive-passiv drainage, Primed medical techniques, Germany). In addition, two Easy Flow drains were placed dorsally to the pancreaticogastrostomy and hepatico-jejunostomy, which are pulled separately left and right through the abdominal wall and fixed to the skin.

### Perioperative Management

Enteral nutrition was administered from the first postoperative day through the alimentary limb of a three-lumen nasogastric tube (Freka® Trelumina FR 16/9, Fesenius Kabi, Germany). Additionally, sips of water were given on the first postoperative day. When there was no clinical evidence of leakage in any of the anastomoses, the enteral feeding flow rate was gradually increased to 70 ml/h. A proton pump inhibitor (pantoprazole, Hexal AG, Germany) was administered during the entire postoperative hospital course. Epidural analgesia was given until 72 h postoperatively. Low molecular weight heparin (Clexane, Sanofi Aventis, Germany) was administered for the prevention of deep vein thrombosis until patients were fully ambulatory.

Several blood values, including serum lipase, protein and albumin, were examined preoperatively. Drainage fluid volume from peripancreatic Easy Flow and pancreatic duct drainage was measured and checked daily. Serum and pancreatic duct-drainage lipase values were measured on postoperative day 3 and 7.

**Figure 3:**
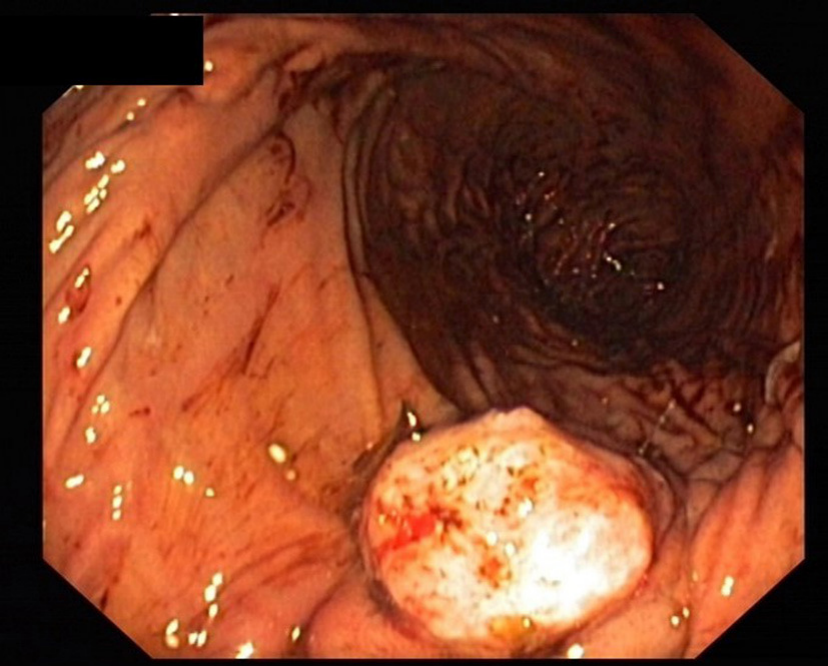
Pancreatic stump after drainage removal.

Peripancreatic Easy Flow drain lipase was measured on postoperative day 3, 5, and 7. The measurement was continued every second day if there were signs of persistent leakage until the drainage was removed. To monitor the inflammatory systemic response, leukocytes and C-reactive protein were measured on the first 3 postoperative days and every third day thereafter (6. /9. /12. etc.). The peripancreatic Easy Flow drains were removed on the 7th postoperative day if there was no evidence of a leakage. However, if there was evidence of leakage or suspected infective complications (fever, leukocytosis and purulent drainage fluid), the peripancreatic drains were left *in situ*. The pancreatic duct drain was removed in most cases between the 6th and 8th postoperative day (Figure 3).

In addition, patients were asked about nausea and vomiting during daily rounds and the abdominal status was examined. All possible relevant complications such as anastomosis bleeding, insufficiency of the hepaticojejunostomy or the entero-enterostomy, delayed gastric emptying and reoperation were assessed.

According to the ISGPS 2016 classification, POPF were classified into 3 groups (5).

- Biochemical leak (former Grade A): is defined as a drain output with a persistent amylase/lipase level, which is 3 times higher or more than the upper limit of normal serum amylase/lipase. Biochemical leak has no clinical relevance.
- Grade B: The Fistula leads to changes in the postoperative Management. The abdominal drain is left for more than 3 weeks or has to be repositioned by endoscopic or percutaneous procedures. It leads to signs of an infection but no organ failure.
- Grade C: Requires a reoperation. It causes systemic infection with single or multiple organ failures up to death.

## Results

The insertion of a suction drain into the pancreas duct in pancreatogastrostomy was feasible in all 21 patients. The drainage of the pancreatic duct was well tolerated by all patients and did not result in increased pain levels. We did not observe any complications or discomfort related to the intrapancreatic duct-drain or delayed gastric emptying.

In addition, no wound infection caused by the pancreatic duct drainage or secretion via the drainage channel were detected after removal. The pancreatic duct drain remained at the mean for 7 days (Table 1). In patients with manifest fistula, the pancreatic duct drainage was removed when the lipase level of peripancreatic Easy Flow liquid was equal to, lower than, or <3 times the lipase value of the serum and the patient was in stable condition without any clinical sign of abdominal pain. In these patients the duct drain was removed between day 15 and 20. We registered drainage dislocation out of the pancreatic duct into the stomach in 4 patients (between day 5 and 7), which was apparent in a change of the quality of the drain fluid.

**Table 1:** Patient demographics, intraoperative data and POPF grading.

	**Gender**	**Age**	**Diagnosis**	**Diameter of the pancreatic drainage (ch)**	**Texture of the pancreatic tissue**	**Po. days with duct drainage**	**POPF**
1	F	75	IPMN main duct	6	Soft	7	–
2	F	82	Leiomyosarkoma of the retroperitoneum	5	Soft	7	–
3	M	64	Adenocarcinoma of the pancreas	5	Soft	7	–
4	F	81	Adenocarcinoma of the pancreas	5	Soft	7	–
5	M	43	Metasis of a rectal cancer	5	Soft	7 (slipped)	B
6	F	76	Adenocarcinoma of the papilla vateri	5	Soft	20	A
7	M	64	Cholangio–carcinoma	4	Middle	8	–
8	F	74	Inflammatory bile duct stenosis	5	Soft	6	–
9	M	72	Cholangio-carcinoma	6	Soft	7	–
10	M	59	Adenocarcinoma of the pancreas	6	Hard	7	–
11	M	67	Adenocarcinoma of the pancreas	6	Soft	15	B
12	W	56	Adenocarcinoma of the pancreas	8	Hard	7	–
13	M	65	Cholangio-carcinoma	6	Soft	8	–
14	M	58	Adenocarcinoma of the pancreas	6	Soft	20	C
15	W	84	Adenocarcinoma of the pancreas	6	Soft	5 (slipped)	–
16	W	62	Adenocarcinoma of the pancreas	8	Hard	7 (slipped)	–
17	W	69	IPMN main duct	8	Soft	13	–
18	M	70	Ampullary tubulovillous Adenoma with HGIEN	5	Soft	7	–
19	M	66	Adenocarcinoma of the pancreas	6	Soft	7 (slipped)	–
20	W	73	Cholangio-carcinoma	6	Middle	7	–
21	W	78	Adenocarcinoma of the Ampulla vateri	6	Soft	7	–

12 of the 16 Patients Had a Soft Pancreas Tissue Texture (Table 1).

The daily secretion volume via the pancreatic duct drainage was between 0 and 240 ml.

Only in one patient the drain did not extract any fluid at all. The fluid of all other patients was clear and the lipase level ranged between 340 and 42,000 U/L (Table 2).

**Table 2:** Lipase level in serum, peripancreatic- and pancreatic duct drainage (in U/l), secretion volume of the pancreatic duct drainage (in ml) and POPF grading.

**Patient**	**Highest lipase level blood in IE**	**Lipase level peripancreatic drainage 7th postoperative day in IE**	**Highest lipase level pancreatic duct drainage in IE**	**Highest daily volume of secretion in ml**	**POPF**
1	76	18	42,000	200	–
2	192	44	4,062	110	–
3	3	10	10,650	15	–
4	13	10	42,000	120	–
5	609	11,809	42,000	150	B
6	41	14,230	32,899	100	A
7	76	10	23,061	50	–
8	62	67	–	0	–
9	3	10	19,898	10	–
10	3	10	42,000	100	–
11	103	42,000	42,000	170	B
12	3	10	340	10	–
13	87	58	42,000	240	–
14	144	6,776	42,000	150	C
15	22	21	4,905	200	–
16	3	10	4,2000	25	–
17	4	40	42,000	75	–
18	115	10	42,000	200	–
19	33	12	42,000	170	–
20	6	10	42,000	130	–
21	21	10	42,000	75	–

**Figure 4:**
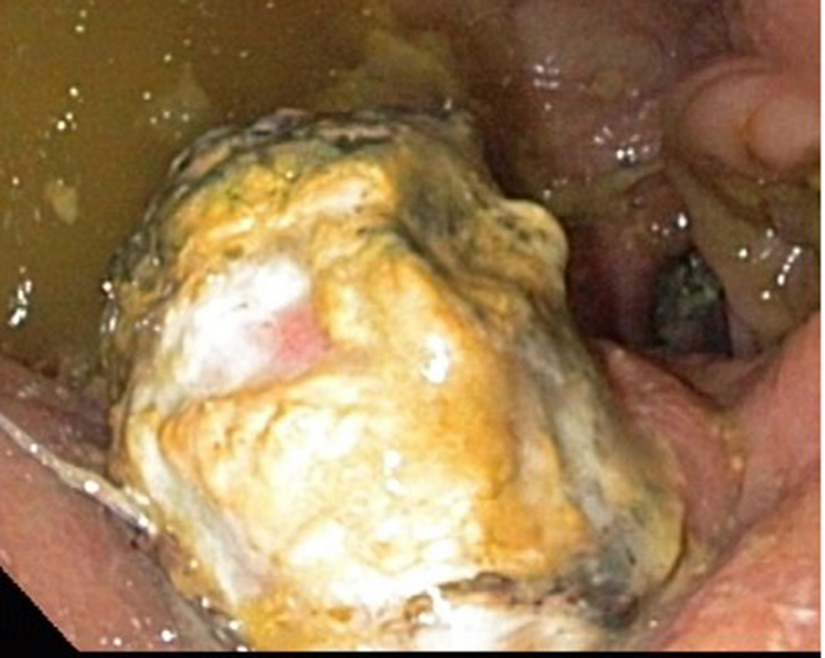
Pancreatic stump of patient 5 (POPF Grade B).

17 of 21 Patients Showed no Signs of POPF. A Biochemical Leak Was Measured in the Case of one Patient. Furthermore, 2 Patients Had a POPF Grade B (Figure 4).

POPF grade C was present in one patient requiring re-operation and resection of the remnant pancreas. Unfortunately, this patient died later in the course of acute heart failure with the secondary medical diagnosis of coronary stenosis.

We further had hemorrhage from the lateral part of the pancreatic resection margin in one patient, which could be treated endoscopically. The bleeding was not related to the pancreatic duct drainage.

**Figure 5:**
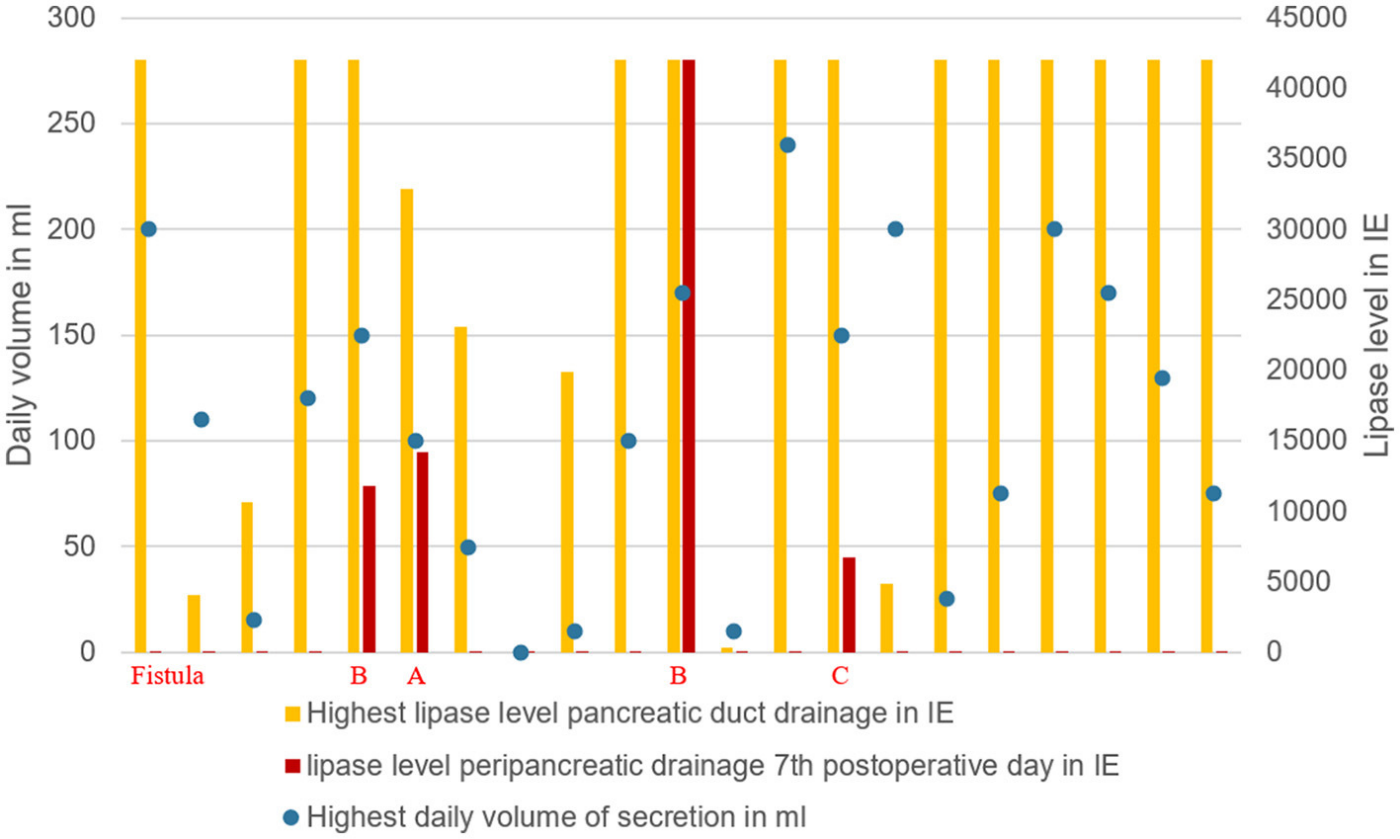
Grade of pancreatic fistula in relation to the highest lipase value and daily secretion volume via pancreatic drainage.

In all 4 cases of POPF, patients had a soft pancreas, a high volume and a high lipase level in the secretion via the pancreatic duct drainage (Table 2; Figure 5).

## Discussion

Advances in pancreatic surgery techniques and perioperative care have resulted in lower pancreaticoduodenectomy (PD) mortality rates in high-volume expert centers (6). However, morbidity after pancreatic resection remains high. Complications occur in 30–60% of patients after surgery, mainly due to leakage and subsequent fistula at the pancreatic anastomosis (7). There are several reports in the literature of the risk factors that could promote anastomotic leakage (2). Most authors agree that two factors play an important role in POPF: first, a soft pancreatic texture and second, an undilated pancreatic duct. Both factors have been most consistently associated with a high rate of POPF (8).

In our study, 16 of the 21 included patients encountered the risk situation of soft pancreatic tissue with a small duct. All 4 cases of POPF were found in these 16 patients of this risk population.

Volume and lipase value in the pancreatic duct drainage secretion had a broad range in our study (Table 2). High volume and high lipase value in the duct drainage seem to indicate a high enzymatic activity of the remnant pancreas. In all 4 cases of POPF the latter criteria were seen (Figure 5).

The use of pancreatic duct stents and drainages has been discussed in the literature, but the published results are still controversial. Table 3 provides an overview of the most important studies investigating the role of negative pressure pancreatic drainage in the prevention of pancreatic fistula. In summary, the use of external negative pressure seems to protect more effectively than external drainage with gravity pressure. All these studies refer only to pancreatojejunostomy (9–12).

**Table 3:** Studies reporting pancreatic duct drainage after pancreaticoduodenectomy with pancreaticojejunostomy compared with our results (PRT prospective randomized trial).

**References**	**Type of study**	**Number of patients**	**Type of drainage**	**Pancreatic fistula rate**	**POPF grade**
(9)	PRT	110 (55 vs. 55)	External, negative pressure vs. gravity pressure	25.5 % vs. 43.6 % (*P* = 0.045)	A: 16.4 vs. 32.7 % B: 5.5 vs. 7.3 % C: 3.6 vs. 3.6%
(10)	PRT	76 (41 vs. 35*)*	External, negative pressure vs. no drainage	69.2 % vs. 70.7 % (*P* = 0.922)	A: 35.9 vs. 13.9 % B: 33.3 vs. 56.8 % p 0.04 C: 0 vs. 0
(11)	Retro-spective	58 (33 vs.25)	External, negative pressure vs. gravity pressure	36.2 % vs. 64 % (*P* = 0.026)	A: 27.2 vs. 24.0 % B + C: 9.0 vs. 40.0 % p 0.012
(12)	Retro-spective	76 (37 vs. 39)	External, negative pressure vs. gravity pressure	9.8 % vs. 31.3 % (*P* = 0.018)	A: 0 vs. 0 % B: 9.8 vs. 14.2 % C: 0 vs. 17.1 %
Gretschel et al. this study	Case series	21	External negative pressure (pancreaticogastrostomy)	19 %	A: 4.8 % B: 9.5 % C: 4.8 %

This study evaluated the implementation of an external pancreatic duct drainage under closed suction with negative pressure in pancreatogastrostomy following PD.

The introduction of a suction drainage into the pancreatic duct was feasible in all patients and did not cause drainage-related morbidity. We registered drainage dislocation into the stomach in 4 patients. This was probably caused by insufficient fixation of the drainage in the soft tissue of pancreas.

Consequently, the correct fixation of the drainage to both the pancreatic stump and the skin must be ensured. One patient with a drainage dislocation developed a POPF grade B.

To what extent the drainage dislocation may have promoted POPF cannot be proven.

The other 3 cases of drainage dislocation did not result in POPF.

In one patient no secretion was found via the pancreatic duct drainage. The phenomenon could have been caused by an incorrect size of the duct drainage or clotted side holes. However, this fact did not affect the regular healing of the anastomosis.

The use of pancreatic ductal drainage did not completely prevent POPF but resulted in a moderate rate of POPF in 4 of 21 patients (19%). In the high-risk population of a soft pancreas with an undilated duct, we saw only one grade C POPF (4.8%) in our feasibility study.

Given the limitations of this feasibility series (limited number of patients, single center study, no control group), however, it cannot be stated to what extent drainage is responsible for the moderate fistula rate in the high-risk population in this study.

The technique is easy to learn and apply and neither leads to a relevant longer operation time nor increases the risk for the patient. It should be emphasized that these are our preliminary results and the first experiences with external drainage under closed suction in pancreatogastrostomy following PD. Our series did not include a control group (no drainage group) and aimed at the evaluation of feasibility of the technique.

## Conclusion

The applied technique of external pancreatic duct drainage under closed suction with negative pressure in pancreatogastrostomy following PD was feasible without any drainage-related risk for the patient. The use of pancreatic duct drainage resulted in a moderate POPF rate in 4 of 21 patients (19%), with only one POPF grade C (4.8%) in the risk population of a soft pancreas with a non-dilated duct. Motivated by the promising results of our feasibility series, we started a prospective randomized study (registration number DRKS00021634) including one arm with no drainage patients to obtain valid data in a larger cohort.

## Data Availability Statement

The original contributions presented in the study are included in the article/supplementary material, further inquiries can be directed to the corresponding author/s.

## Ethics Statement

The studies involving human participants were reviewed and approved by Ethics Committee of Brandenburg Medical School (08 April 2019/No. E-01-20181120). The patients/participants provided their written informed consent to participate in this study.

## Author Contributions

HA, CA, and CM collected and analyzed the data and wrote main parts of the manuscript. SG and HA designed the study and completed the manuscript. All authors meet the criteria of the International Committee of Medical Journal Editors (ICMJE) regarding the definition of authorship.

## Conflict of Interest

The authors declare that the research was conducted in the absence of any commercial or financial relationships that could be construed as a potential conflict of interest.

## Publisher's Note

All claims expressed in this article are solely those of the authors and do not necessarily represent those of their affiliated organizations, or those of the publisher, the editors and the reviewers. Any product that may be evaluated in this article, or claim that may be made by its manufacturer, is not guaranteed or endorsed by the publisher.
